# The Changes in Postural Stability of Women in Early Old Age

**DOI:** 10.1007/s12603-020-1399-z

**Published:** 2020-05-29

**Authors:** G. Olchowik, A. Czwalik, Bartłomiej Kowalczyk

**Affiliations:** 1 Chair and Department of Biophysics, Medical University of Lublin, ul. Jaczewskiego 4, 20-090, Lublin, Poland

**Keywords:** Balance system, computerised dynamic posturography, older women, postural stability, motor strategy

## Abstract

**Background:**

Age is one of the most frequent reasons mentioned for the deterioration in human postural stability. Involution processes associated with a deterioration of stability are primarily: a slowing of the motor reactions related to a decline in the average conduction rate of nerve impulses, loss of muscle mass, loss of receptors in the balance controlling organs, and a decrease in visual acuity. The aim of this study was to determine in which organ, responsible for controlling human balance, changes will already appear in early old age, and whether these changes can be diagnosed using CDP.

**Methods:**

The study was conducted on a group of 141 women (41 elderly aged 65.5 ± 4.6 years, 100 young women aged 20.7 ± 1.2 years). The posturographic study was carried out using the dynamic EquiTest posturograph manufactured by NeuroCom International. The study protocol included the Sensory Organisation Test (SOT), the Motor Control Test (MCT) and the Adaptation Test (ADT).

**Results:**

The SOT results show significantly greater displacements of the body's Centre of Gravity projected onto the posture plane in the anterior-posterior direction in the elderly under all test conditions. The Latencies, determined from MCT, differed significantly between the two groups under all test conditions.

**Conclusion:**

In women aged over 60, the perception of stimuli received by the visual and vestibular organs is impaired and their proficiency in controlling body balance is lower. Thus, they are more likely to adopt a hip strategy to maintain balance.

## Introduction

In literature, age is one of the most frequent reasons mentioned for the deterioration in human postural stability. Involution processes associated with a deterioration of stability are primarily: a slowing of the motor reactions related to a decline in the average conduction rate of nerve impulses, loss of muscle mass, loss of receptors in the balance controlling organs, and a decrease in visual acuity (1, 2). In the aging process the perception of all stimuli received by the sense organs responsible for maintaining balance is impaired (3). These factors gradually reduce the area of stability of a standing posture, which results in an increased risk of falls among the elderly. Falls in a group of elderly people are associated with many serious consequences such as fractures, disability, or even death and are more frequent than in other age group (4).

An assessment of the functioning status of the balance control system can be carried out using many clinical tests, but not all of the tests are able to differentiate the patients' results quantitatively. Research conducted by the authors of studies (5, 6, 7) indicate that it is justifiable to use Computerised Dynamic Posturography (CDP) for a quantitative and qualitative assessment of the human balance system. The main parameter controlled during posturographic examinations is the location and speed of displacement of the Centre of Gravity (COG) projection onto the posture plane. Based on the CDP research protocol the following are assessed: the effectiveness of the sensory organs involved in postural control; the correctness of the selection of an appropriate motor strategy; as well as the latency and amplitude of the postural response.

The aim of this study was to determine in which organ, responsible for controlling human balance, changes will already appear in early old age, and whether these changes can be diagnosed using CDP. People in early old age were selected for the study group because, in the sixth decade of life, changes in the balance system emerge (8).

## Materials and Methods

The study was conducted on a group of 141 women (41 elderly aged 65.5 ± 4.6 years, 100 young women aged 20.7 ± 1.2 years).

The study involved subjects with none of the following: postural defects, dizziness, hearing loss, tinnitus, or history of ear infections, as well as those who in the past, did not experience serious head or spinal trauma, were not treated for chronic diseases, and did not take medication during the previous month that could impair the functioning of the balance system.

The posturographic study was carried out using the dynamic EquiTest posturograph manufactured by NeuroCom International. The study protocol included the Sensory Organisation Test (SOT), the Motor Control Test (MCT) and the Adaptation Test (ADT).

SOT was performed using six sensory stimulation conditions (Table 1) (9).

**Table 1:** Sensory stimulation conditions of the sensory organization test

**Test/Condition**	**Vision**	**Foot Support Platform**	**Visual Surround**
SOT 1	Eyes Open	Stationary	Stationary
SOT 2	Eyes Closed	Stationary	Stationary
SOT 3	Eyes Open	Stationary	Moving
SOT 4	Eyes Open	Moving	Stationary
SOT 5	Eyes Closed	Moving	Stationary
SOT 6	Eyes Open	Moving	Moving

As part of SOT the following analysis were performed: Equilibrium Score (ES) and Motor Strategy (MS) (10). The ES result provides a quantitative assessment of the displacement of the body's COG in the anterior-posterior direction (11). ES values are presented as a percentage. A value of 100% means no displacement of the body's COG in the anterior-posterior direction. The greater this displacement, the lower the ES value. The Composite Equilibrium Score (CES) which is an indicator of overall body balance takes into account the displacement of the body's COG under all SOT conditions (12).

The Sensory Analysis (SRS) results are presented separately for the Somatosensory System (SOM), the Visual System (VIS), the Vestibular System (VES) and Visual Preference (PREF). They affirm the usefulness of the signal from a given sensory system in maintaining body balance. They are presented as percentages, A value of 100% means full proficiency of a given sensory system and the correct use of the signal from this system in controlling body balance.

MS analysis enables a quantitative assessment of muscle activity responsible for movements in ankle and hip joints during each SOT test. The higher the MS value, the greater the muscle activity responsible for movements in the ankle joints compared to muscle activity responsible for movements in the hip joints.

MCT was performed for 6 conditions using the foot support platform capable of forward and background movements through small, medium and large displacements. The patient's ability to perform corrective movements in response to unexpected perturbations of the foot support platform was assessed. The visual surround is stationary. For MCT, the analysed parameters were the Latency Response (L) and the Amplitude (A) of the postural response, expressed in degrees per second. L is the time delay (milliseconds) between the start of platform movement and the start of the postural response of both lower limbs. In addition, the symmetry of body weight distribution, enabled a quantitative assessment of the load on both lower limbs to be made (10, 11). Based on these values, the Total Postural Response Latency (LC) score was calculated.

For ADT, the subject is subjected to five abrupt platform perturbations, resulting in either flexion or extension of the ankle joints. With each subsequent attempt, the subject should maintain a vertical posture thus minimising, for each subsequent attempt, the amount of energy needed to return the body to equilibrium. Sway Energy (SE) was the analysed parameter. It is a dimensionless quantity in the range from 0 to 200 which indicates the magnitude of COG displacement during each test. The greater the body's COG displacement during return to the equilibrium position, the greater the body's imbalance caused by the foot support platform (10, 11).

Statistical analysis was performed using the STATISTICA 10 (StatSoft) computer program. Statistically significant correlations were accepted where significance level p<0.05.

## Results

The SOT results are presented in Table 2. The results show significantly greater displacements of the body's COG projected onto the posture plane in the anterior-posterior direction in the elderly under all test conditions. Increased COG oscillations were also found in the elderly due to disorders in position control. Statistically significant differences between the two groups were also found in the proficiencies of the vestibular and visual organs (Table 2). In both cases, the elderly achieved results indicating a lower organ proficiency in balance control. The study groups differed statistically significantly in the selection of a motor strategy (MS) (Table 2). Lower values in the group of elderly women indicate a predominance of muscle activity responsible for movement in the hip joints.

**Table 2:** The results of postural response delay analysis of the left (LL) and right (LR) lower limbs, total response delay (LC) and the results of the analysis of the amplitude of the left (AL) and right (AR) response of the lower limb to load plate displacement in the motor control test (MCT) in a group of seniors and young women

	**Seniors**	**Young women**	**p**	**I**
	**A(±SD)**	**A(±SD)**		
ES1 [%]	92,4(±2,8)	94,1(±2,3)	0,0007	*
ES2 [%]	91,8(±3,1)	93,6(±2,1)	0,0008	*
ES3 [%]	89,2(±4,8)	92,1(±3,2)	0,0013	*
ES4 [%]	76,6(±7,0)	83,7(±6,6)	<0,0001	*
ES5 [%]	62,5(±9,4)	70,1(±7,6)	<0,0001	*
ES6 [%]	58,0(±10,3)	65,6(±10,3)	0,0002	*
CES [%]	73,4(±6,4)	80,2(±4,6)	<0,0001	*
SOM [%]	98,8(±1,8)	98,9(±1,4)	0,7595	ns
VIS [%]	82,6(±7,8)	89,0(±6,7)	<0,0001	*
VEST [%]	67,4(±11,2)	74,5(±7,9)	0,0007	*
PREF [%]	94,1(±7,1)	95,3(±5,0)	0,7441	ns
MS1 [%]	94,0(±3,1)	95,8(±1,9)	0,0002	*
MS2 [%]	93,9(±3,5)	95,8(±1,5)	0,0014	*
MS3 [%]	90,1(±7,7)	95,0(±2,4)	<0,0001	*
MS4 [%]	82,4(±7,4)	90,7(±4,1)	<0,0001	*
MS5 [%]	72,8(±10,7)	83,1(±6,5)	<0,0001	*
MS6 [%]	69,4(±11,9)	82,1(±7,6)	<0,0001	*


Arithmetic mean (A), standard deviation (SD), significance factor (p), level of statistical significance (I): p <0.05 - ‘*â€™,p≥0.05 as not statistically significant - ‘nâ€™

The Latencies (L), determined from MCT, differed significantly between the two groups under all test conditions, which was reflected in significant differences in the overall result of LC (Table 3). The elderly responded to the foot support platform perturbations much more slowly than the young.

**Table 3:** The results of body balance analysis (ES), motor strategy (MS) and sensory analysis (SRS): somatosensory system (SOM), sight organ (VIS), vestibular organ (VEST) and visual preference (PREF) in sensory organization test (SOT) and the total result of balance analysis (CES) in the group of seniors and young women

	**Seniors**	**Young women**	**p**	**I**
	**A(±SD)**	**A(±SD)**		
LLSBT [ms]	158,3(±18,9)	139,3(±13,0)	<0,0001	*
LLMBT [ms]	148,5(±14,9)	129,9(±11,4)	<0,0001	*
LLLBT [ms]	142,7(±12,6)	122,3(±10,5)	<0,0001	*
LLSFT [ms]	153,9(±18,3)	138,1(±14,8)	<0,0001	*
LLMFT [ms]	149,5(±16,9)	134,3(±14,1)	<0,0001	*
LLLFT [ms]	142,4(±13,0)	126,0(±10,9)	<0,0001	*
LRSBT [ms]	157,6(±16,7)	137,0(±12,4)	<0,0001	*
LRMBT [ms]	145,6(±15,8)	126,3(±12,3)	<0,0001	*
LRLBT [ms]	137,6(±11,6)	119,5(±9,7)	<0,0001	*
LRSFT [ms]	153,4(±16,8)	135,3(±14,6)	<0,0001	*
LRMFT [ms]	145,6(±16,3)	131,4(±12,4)	<0,0001	*
LRLFT [ms]	136,3(±12,6)	123,8(±11,0)	<0,0001	*
LC [ms]	143,6(±11,2)	126,9(±7,9)	<0,0001	*
ALSBT [o/s]	2,8(±1,6)	2,9(±1,7)	0,7458	ns
ALMBT [o/s]	5,3(±2,1)	5,5(±2,6)	0,9784	ns
ALLBT [o/s]	7,4(±2,5)	8,7(±3,3)	0,0605	ns
ALSFT [o/s]	3,0(±1,7)	2,9(±1,5)	0,6252	ns
ALMFT [o/s]	6,0(±2,4)	5,7(±2,2)	0,4896	ns
ALLFT [o/s]	7,8(±2,3)	8,1(±2,3)	0,3837	ns
ARSBT [o/s]	3,0(±1,7)	2,9(±1,6)	0,6526	ns
ARMBT [o/s]	5,6(±2,3)	5,6(±2,6)	0,6837	ns
ARLBT [o/s]	7,7(±2,4)	8,8(±3,6)	0,2455	ns
ARSFT [o/s]	3,4(±1,7)	3,0(±1,4)	0,2643	ns
ARMFT [o/s]	6,4(±2,2)	6,0(±2,2)	0,3286	ns
ARLFT [o/s]	8,0(±2,4)	8,5(±2,5)	0,1321	ns


Arithmetic mean (A), standard deviation (SD), significance factor (p), level of statistical significance (I): p <0.05 - ‘*â€™,p≥0.05 as not statistically significant - ‘nâ€™

There were no significant differences in the amplitude response (A) between the two groups, which indicates a similar angular velocity conveyed to the COG during recovery, regardless of the subjects' age.

The ADT results which assess the subjects' adaptation mechanisms for cyclically repeated foot support platform perturbations by age are presented in Table 4. The SE results differed significantly between the two groups during ankle flexion (first and second attempt) and during ankle extension (second, third and fourth attempt). Higher values were observed in the elderly, which testifies to a lower proficiency of their adaptation mechanisms in response to repeated perturbations of the foot support platform.

**Table 4:** The results of the swinging energy analysis (SE) in the adaptation test (ADT) in a series of five tests causing flexion (ATU1-ATU5) and extension (ATD1-ATD5) in ankles in the group of seniors and young women

	**Seniors**	**Young women**	**p**	**I**
	**A(±SD)**	**A(±SD)**		
SE ATU1	74,4(±15,3)	86,8(±17,3)	0,0034	*
SE ATU2	66,7(±15,5)	75,2(±15,4)	0,0114	*
SE ATU3	62,5(±14,6)	68,2(±14,5)	0,0707	ns
SE ATU4	61,3(±15,2)	63,3(±10,1)	0,1465	ns
SE ATU5	62,4(±13,5)	61,4(±10,2)	0,9283	ns
SE ATD1	60,3(±19,8)	54,4(±13,4)	0,1183	ns
SE ATD2	56,8(±19,1)	45,7(±11,8)	0,0069	*
SE ATD3	49,6(±14,9)	42,6(±9,8)	0,0211	*
SE ATD4	50,4(±16,7)	41,0(±8,7)	0,0005	*
SE ATD5	45,3(±13,2)	39,9(±8,4)	0,0592	ns


Arithmetic mean (A), standard deviation (SD), significance factor (p), level of statistical significance (I): p <0.05 - ‘*â€™,p≥0.05 as not statistically significant - ‘nâ€™

## Discussion

The SE differences between the two groups show that the elderly are less stable in the anterior-posterior direction, regardless of the availability and accuracy of the sensory information reaching the body. The study authors (13) believe that it is instability in the frontal plane that is the main cause of imbalance and consequently, falls in the elderly.

The SRS results indicate an impairment of stimuli perception received by the visual and vestibular organs, as well as their lower proficiency in controlling body balance in elderly women. (Table 2). This result indicates the necessity to monitor and appropriately correct eyesight early enough in order to limit the already weakened by age postural stability. Deteriorated vision also has an impact restricting activity and consequently, physical fitness. The important role of visual information is also confirmed by study (14). In addition, the authors of study (8) observed a significantly larger sway area in the absence of a visual signal and better test results un the presence of a visual signal, which emphasises its huge role in the process of maintaining balance. Faraldo-García et al, (15) observed a decrease in the usefulness of visual information up to about the age of 50, followed by its increase, with an accompanying decrease in the usefulness of vestibular information, The increase in the importance of visual information after the age of 50 was caused by the introduction of vision correction (spectacles, contact lenses) and better use of visual stimuli. Worse use of information emanating from the vestibular organ in controlling body balance in the elderly is probably related to progressive structural changes during the aging process, such as the reduction in the number of vestibular nerve fibres or a reduction in the sensory epithelial cell population.

The differences in the selection of the motor strategy, with a hip strategy predominance in elderly women, are confirmed by Liaw et al. (16).

The postural response time results (L) obtained in the MCT showed a longer Latency of 9.8% in women in early old age. Mackey and Robinovitch study confirm these results in the elderly (17). The authors observed a 27% increase in the reaction time in a group of elders aged 66–90, which in their opinion was also associated with weaker muscle strength due to age. Perrin et al. also arrived at a similar conclusion (18). However, after height normalisation, the extended latency results in the studies of Peterka and Black (19) turned out to be statistically insignificant. The authors suggest, that the prolonged response time is due to the subjects' greater height resulting in a longer nerve impulse conduction path. However, our research results indicate that it is not height, but age which is the cause of a prolonged response time to unexpected foot support platform perturbations, because the older women were significantly shorter than the young. In the tests, no significant postural response amplitude (A) differences were found between the two groups under any MCT condition. This means that, in the period of early old age, age does not significantly affect the amount of angular velocity that a given postural response gives to the body's COG. The results for both the young and the old indicated identical loading on both lower limbs, without a clear trend of overburdening one of them. Therefore, it was decided not to include the symmetry of lower limb loading results in this paper. From a biomechanical point of view, the most effective, and with equal probability of regaining a stable posture, is the silhouette with the body weight evenly distributed between the lower limbs.

The authors of studies (13, 20) suggest that the elderly in the twentieth century assume a preventive asymmetrical stance, since relieving one of the limbs reduces the time required to take a step in the event of a sudden perturbation of the foot support platform.

However, study authors (21) conducting research on people without any pathological symptoms from different age groups claim that it is the speed and amplitude of the body's COG projected onto the base plane in the anterior-posterior direction that are the most differentiating parameters for the age groups. According to the authors, the most useful parameter in identifying the causes of body instability, is the SOT5 result; the test is performed with an incorrect somatosensory signal and lack of visual information. The rapidity of swaying in the anterior-posterior direction is also considered by study authors (22) to be the best prognostic factor for falls in the in the elderly. The authors conclude that thanks to periodic review of postural parameters, the extent of postural control impairment can be determined quite precisely, which may be helpful in distinguishing between the causes related to the aging process and those related to neurodegenerative diseases. Wallmann (23) also presents the rationale for using CDP for a risk assessment of falls in the elderly.

The aging process affects almost all the organs within the human body. Some changes, such as osteopenia (bone mineral density loss), a precursor to osteoporosis, significantly influence the risk of serious consequences resulting from falls in the elderly (24, 25). In order to reduce the risk of fractures and relieve the skeletal system, it is extremely important for people in advanced age to remain in good physical condition. Correct body posture, muscle strength, and better motor coordination are only part of the benefits that can be gained through properly selected training. Konrad, Girardi and Helfert (26), dealing with the problem of deteriorated posture stability among the elderly, emphasise the importance of an appropriately early rehabilitation intervention and the introduction of regular physical exercises to improve the physical condition in the elderly. Fabris de Souza et al. (27) also emphasise the importance of planned physical activity during the rehabilitation process aimed at improving the process of regaining balance and preventing falls.

Unstable body posture and the resultant falls are common in geriatric patients (28). Falls are one of the main causes of injuries and hospitalisations, carrying with them not only medical but also economic and social consequences. Even minor falls in the elderly can carry with them serious consequences in the form of injuries, because of frequent physiological changes due to body aging as well as pathological changes in the nervous system. These result in a deterioration in the quality of defensive reflexes in addition to diseases which weaken the locomotor system. Therefore, early detection of balance disorders and the implementation of appropriate rehabilitation programs aimed at improving balance is extremely important (29, 30).

## Conclusions

In women aged over 60, the perception of stimuli received by the visual and vestibular organs is impaired and their proficiency in controlling body balance is lower. Thus, they are more likely to adopt a hip strategy to maintain balance.

In early old age, regardless of the availability and correctness of sensory information, there is greater body sway in the anterior-posterior direction, while the response time to perturbations of the foot support platform is longer.

Thanks to periodic reviews of postural parameters diagnosed with CDP, the degree of impairment of postural control can be determined quite precisely. This can be helpful in distinguishing between causes related to the aging process and causes related to neurodegenerative diseases.
